# Interacting roles of gut microbiota and T cells in the development of autoimmune hepatitis

**DOI:** 10.3389/fimmu.2025.1584001

**Published:** 2025-05-26

**Authors:** Qingwei Wu, Zhifa Ge, Chengyu Lv, Qifeng He

**Affiliations:** Department of General Surgery, Nanjing First Hospital, The Affiliated Nanjing Hospital of Nanjing Medical University, Nanjing, Jiangsu, China

**Keywords:** gut microbiota, autoimmune hepatitis, T cells, interaction, immune microenvironment

## Abstract

Autoimmune hepatitis (AIH) is a progressive liver inflammatory disease mediated by an autoimmune response, with an increasing incidence rate. In severe cases, AIH will rapidly progress to liver cirrhosis and liver failure and even lead to death. The gut microbiota is a complex ecosystem that significantly regulates physiological and pathological processes among various digestive system diseases. It is widely acknowledged that there is a critical correlation between AIH and the gut microbiota. Numerous studies have demonstrated that the composition of gut microbiota in individuals with AIH differs markedly from that of healthy subjects. Immune cells, especially T cells, are pivotal in the development of AIH, closely interacting with the gut microbiota. In this review, we discuss the regulatory role of the gut microbiota in T cell-mediated development of AIH, as well as the effect of T cells on the composition of the gut microbiota in AIH. By modulating gut microbiota or immunity pathways, novel opportunities are provided to regulate the balance of the immune-microbial microenvironment, targeting the dual factor for autoimmune hepatitis therapies.

## Introduction

In recent years, there has been an increase in the incidence of autoimmune diseases such as autoimmune hepatitis (AIH) and inflammatory bowel disease (IBD), type 1 diabetes (T1D), rheumatoid arthritis (RA), and multiple sclerosis (MS), attributed to changes in lifestyle and environmental factors (1, 2). In exploring the occurrence of autoimmune diseases, there is an amounting recognition of the metagenome of all colonized microorganisms in the body, known as the microbiome (3). Although both the microbial and host genomes are regulated by diet and environmental factors, the microbial genome exhibits a rapid adaptability to environmental shifts due to its shorter duplication cycles. Therefore, pieces of evidence support that gut microbiota plays an essential role in autoimmune diseases, which regulates the taxonomic composition of microbial communities and their metagenomic functions (4).

The co-evolution of the immune system and microbiota has been identified to participate in complex immune decision-making processes. The immune system communicates with the microbiota through several recognition patterns, among which T cells possess the most extensive microbial recognition capability, ranging from detecting the entire microbial community to recognizing specific antigens. These microorganisms are not only an important source of antigen variation but also provide necessary signals for the normal development, maturation, and epigenetic modulation of T cells, while mature T cells regulate microbial responses (5). Furthermore, gut microbiota dysbiosis can also regulate liver pathophysiology and induce AIH (6).

AIH is a serious and progressive autoimmune disease characterized by high levels of gamma globulin and hepatic inflammatory infiltration. If not timely diagnosed and treated, AIH increases the risk of liver cirrhosis and end-stage liver failure (7). Although the exact pathogenesis of AIH remains unclear, studies indicate that genetic factors, environmental factors, and aberrant immunological regulatory mechanisms are involved (8). The mechanisms by which the gut microbiota influences AIH involve multiple levels, including gut microecological imbalance, which leads to the production of autoantibodies and hepatocyte damage (9). The degree of dysbiosis correlates with the severity of liver inflammation, and changes in microbial metabolites affect the hepatic immune microenvironment through the gut-liver axis (10). Furthermore, individuals with AIH frequently exhibit impaired intestinal barrier integrity, facilitating the transfer of gut bacteria or their structural components and metabolites to the liver, which activates innate immune responses, thereby exacerbating liver inflammation and autoimmune reactions (11). Alterations in the gut microbiota regulate the composition and circulation of short-chain fatty acids and bile acids, directly impacting the liver’s metabolic and inflammatory states (12). However, the specific mechanisms by which the gut microbiota and their metabolites are involved in the development of AIH have not been fully elucidated. More clinical and basic studies are being dedicated to improving AIH therapy through the gut microbiota (13).

This review discusses the roles of gut microbiota and their metabolites in the regulation of T cells during the development of AIH, as well as how T cells, in turn, regulate the composition of the gut microbiota within the AIH microenvironment. By modulating the gut microbiota, it is possible to modify the characteristics and functions of immune T cells, providing novel potential targets for the treatment of AIH.

## The gut microbiota: an indispensable player in AIH

The gut microbiota, when continuously challenged by external dietary and environmental antigens, maintain normal physiological functions and dynamic equilibrium within the body (14). In normal circumstances, these microorganisms play a critical role in maintaining the structure of the gut, enhancing barrier integrity, and regulating mucosal immune responses by preserving cellular junctions and promoting epithelial repair (15). Changes in gut permeability and bacterial translocation in AIH patients are associated with disease progression (16). A recent study indicates that the gut microbiota and their metabolites, as a reservoir of exogenous antigens, are crucial for maintaining liver immune homeostasis (17). For instance, *Akkermansia muciniphila* enhances intestinal barrier integrity by degrading mucins within the intestinal mucus layer, thereby stimulating mucin renewal (18). This barrier-protective mechanism effectively reduces the translocation of pathogenic organisms and bacterial metabolites. Consequently, it mitigates hepatic immune activation triggered by gut-derived inflammatory signals, while preserving hepatic immune tolerance (19). HBXN2020, isolated from healthy black pigs, is a microorganism with potent stress resistance and broad-spectrum antibacterial activity. HBXN2020 significantly modulates cytokine levels and maintains the expression of tight junction proteins and mucin proteins, thereby enhancing the stability of gut microbiota and increasing the population of beneficial bacteria (20). Nicotinamide adenine dinucleotide (NAD) activates the ARTC2/P2RX7 pathway *in vivo* and *in vitro*, depleting P2RX7-sensitive unconventional T cells (21). Succinic acid improves ConA-induced liver injury by modulating immune balance, inhibiting pro-inflammatory factors, and promoting anti-apoptotic proteins in the liver (22). To gain an intensive understanding of the composition and relative abundance of the gut microbiota, 16S rRNA gene sequencing has been widely used to characterize these microbiomes and infer the pathogenesis of AIH. Studies have revealed significant changes in the gut microbiota abundance of AIH patients and animal models, including decreased bacterial diversity and increased relative abundance of aerobic or facultative anaerobic microorganisms (23, 24).

Dysbiosis of the gut microbiota is characterized by an imbalance in microbial composition or function, such as reduced diversity, abnormal abundance of specific microbial taxa, or altered metabolic activity. When this imbalance exceeds the host’s capacity for self-regulation, it may lead to inflammation, metabolic disorders, or immune dysfunctions. In patients with AIH, both alpha diversity and beta diversity of the gut microbiota are significantly reduced. The gut microbiota richness, as measured by the Chao1 index, and evenness, as measured by the Shannon index, in AIH patients is much lower than that of healthy controls. The decline in diversity is especially evident during active disease phases or in patients with abdominal symptoms (25). The composition of the gut microbiota in AIH patients shows significant differences compared to healthy individuals, indicating a disruption in the overall microbial structure (26). There is a notable decrease in beneficial bacteria within the gut microbiota of AIH patients, such as a significant reduction in the abundance of *Bifidobacterium* and *Faecalibacterium*, which are usually associated with anti-inflammatory effects and intestinal barrier functions (27). Conversely, there is an increase in potential pathogenic bacteria in AIH patients, with genera such as *Veillonella*, *Streptococcus*, and *Lactobacillus* overgrowing, potentially exacerbating liver damage through immune activation or production of pro-inflammatory metabolites (27, 28). The decreased diversity of the gut microbiota in AIH patients may also be accompanied by reductions in functional genes involved in the metabolism of short-chain fatty acids, amino acid synthesis, and vitamin metabolism, thereby affecting the host’s immune and metabolic functions (26). The etiology of gut microbiota dysbiosis in AIH patients arises from intricate interactions among immunological dysregulation, metabolic disturbances, and environmental factors (**Table 1**).

**Table 1:** Potential causes and mechanisms of gut microbiota dysbiosis in AIH.

Category	Specific causes and mechanisms	Potential impact on AIH	Reference
Diet and metabolism	High-fat/high-sugar diets	Reducing microbial diversity and compromising immune tolerance	(29, 30)
	Vitamin D deficiency		
Medications	Long-term immunosuppressants	Indirect alteration of microbiota composition	(31, 32)
	Antibiotic overuse	Reducing beneficial taxa and increasing pathobiont colonization	
Immune dysregulation	Autoimmune hyperactivation	A vicious cycle between microbiota dysbiosis and immune dysfunction	(33)
Genetic susceptibility	HLA-DR3/DR4 haplotypes potentially altering host-microbiota interactions	Enhancing sensitivity to dysbiosis and increasing AIH susceptibility	(34, 35)
Other triggers	Coexisting autoimmune disorders	Synergistic disruption of microbiota-immune homeostasis	(36, 37)
	Environmental toxin exposure		

### The involvement of gut microbiota and its metabolites in the development of AIH

**Figure 1:**
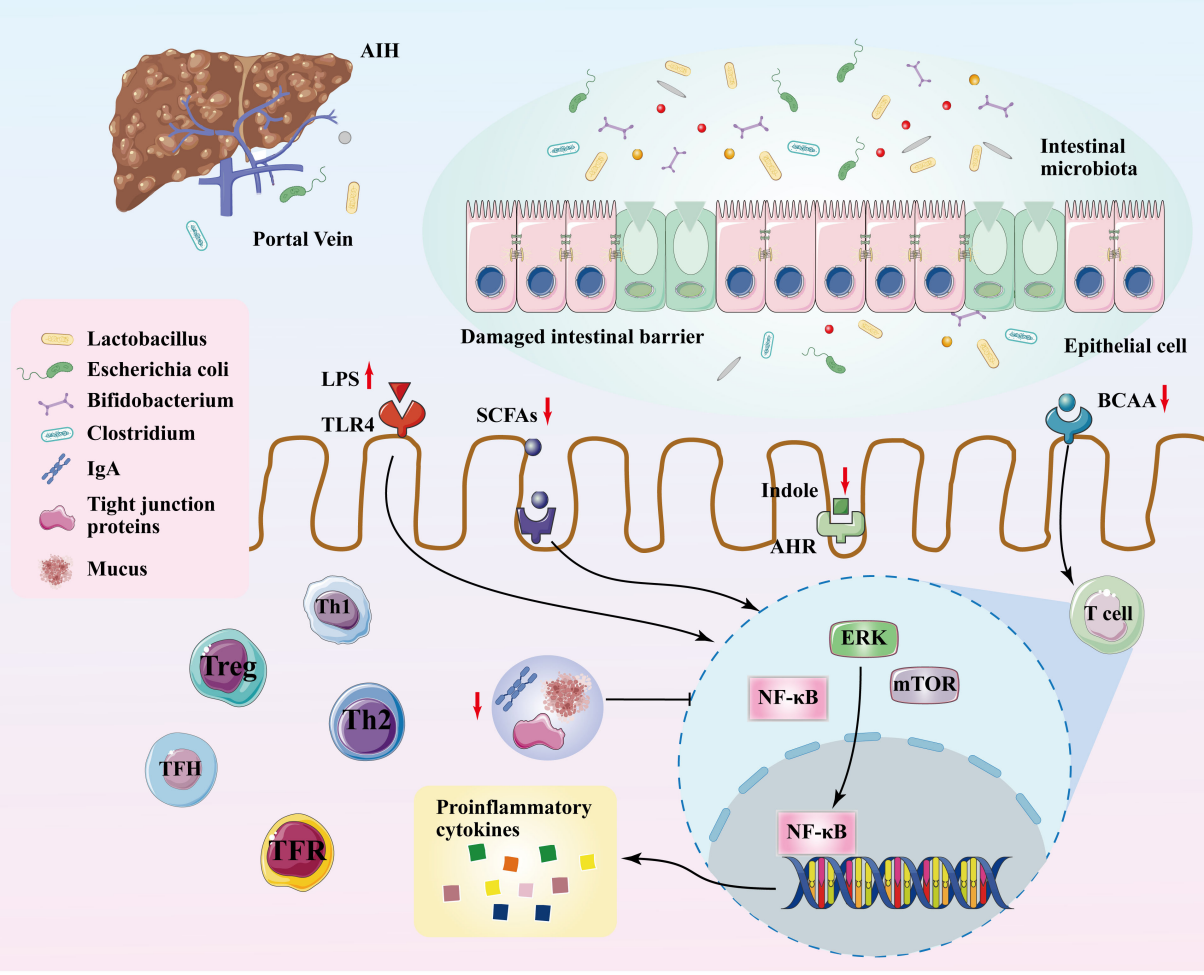
Mechanisms of gut microbiota involved in the development of AIH. Translocation of gut microbiota, alterations in microbial metabolism, and the destabilization of immune homeostasis play crucial roles in the development of AIH. Dysbiosis in the gut microbiome leads to changes in metabolic products and breaks tight junctions of the intestinal barrier. Gut microbiota migrates to the liver and exacerbates hepatic inflammation. Alterations in microbial metabolites stimulate ERK and mTOR, activating the NF-κB signaling pathway, which triggers the expression of inflammatory cytokines and enhances the response of immune cells. Changes in the gut microbiome and its metabolites disrupt the balance among immune cells such as Treg, Th17, Th1, Th2, TFH, and TFR. AIH, autoimmune hepatitis; ERK, extracellular regulated protein kinases; mTOR, mammalian target of rapamycin; NF-kB, nuclear factor-kappa B; Treg, regulatory T; Th17, T helper 17; Th1, T helper 1; Th2, T helper 2; TFH, follicular helper T; TFR, follicular regulatory T; LPS, lipopolysaccharide; SCFAs, short-chain fatty acids; AHR, arylhydrocarbon receptor; BCAA, branched-chain amino acids; TLR4, toll-like receptor 4.

The pathophysiology of AIH is far from being fully clarified. However, current studies have indicated that the gut microbiota and its metabolites play a significant role in the development of AIH (**Figure 1**). These microorganisms modulate immune responses in both the gut and liver through specific signaling pathways. For example, disruptions in the gut microbiota interfere with the WNT/β-catenin signaling pathway, resulting in damage to the gut vascular barrier (GVB) and facilitating bacterial translocation to the liver (38). Impairment of intestinal barrier function and bacterial translocation activates the NLRP3 inflammasome in the liver, leading to inflammatory responses (39). HIF-1α plays a crucial role in the transcriptional regulation of intestinal barrier integrity and inflammation and is essential for maintaining gut microbiota homeostasis and the integrity of the intestinal barrier (40). Dysbiosis of the gut microbiota disrupts the intestinal barrier, leading to the translocation of microbes to the liver (41). Acetylated bacterial lipoproteins are integral components of bacterial cell walls, increasing when the gut microbiota balance is disrupted, thus exacerbating the immune response of the liver (42). Double-stranded DNA represents the genetic material of certain bacteria, recognized by antigen-presenting cells (APCs) in the liver. The recognition of double-stranded DNA triggers the activation of the NF-κB and Mitogen-Activated Protein Kinase (MAPK) pathways, thereby promoting the release of pro-inflammatory cytokines (42). In the liver, lipopolysaccharides (LPS) can activate hepatocytes and hepatic stellate cells (HSCs) through the TLR4-mediated signaling pathway. The activation of hepatocytes and HSCs results in the production of inflammatory and fibrogenic factors, thereby promoting the progression of liver injury and fibrosis (43). An excess of gut microbial toxins abnormally activates the innate immune system and triggers signaling pathways associated with hepatic inflammatory response, thereby disrupting liver homeostasis (23). Due to damage to the intestinal barrier and immune homeostasis imbalance, the gut microbiota continuously act as antigens, initiating and maintaining the autoimmune response in AIH (44).

The imbalance of gut microbiota regulates the production of metabolites within the gut, including short-chain fatty acids (SCFAs), amino acids, and bile acids. These changes alter the permeability and integrity of the gut barrier and the immune homeostasis of the body. The increase of SCFAs lowers the pH in the gut, thereby inhibiting the growth of pathogens (45), which helps restore the imbalanced gut microbiota in AIH. Studies have demonstrated that SCFAs reduce T helper 1 (Th1) cells and increase the number of Treg cells, thus alleviating inflammatory responses in multiple autoimmune diseases. This suggests that SCFAs relieve inflammatory damage in AIH (46). Additionally, the main components of SCFAs include acetate, propionate, and butyrate, among which butyrate plays a key role in enhancing mucin expression in intestinal epithelial cells (IECs), improving the integrity of tight junctions and altering bacterial adhesion (47). At the same time, butyrate lowers levels of pro-inflammatory factors induced by LPS, such as tumor necrosis factor-α (TNF-α), interleukin (IL)-1β, and IL-6, while promoting the secretion of anti-inflammatory factors like IL-10 (48). Therefore, the decrease in SCFAs appears to be related to the disruption of gut barrier function and immune homeostasis, potentially accelerating the progression of AIH. Interestingly, while SCFAs suppress T cell-mediated adaptive immunity, they enhance inflammation induced by innate immune cells. For instance, SCFAs ameliorate the disease severity in collagen-induced arthritis (CIA), yet exacerbate disease severity in antigen-induced arthritis (AIA). The CIA model requires both adaptive and innate immune responses to induce the disease, where the balance among pathogenic Th1 and T helper 17 (Th17) cells, as well as Treg cells, plays a role in inhibiting disease progression. In contrast, AIA does not require adaptive immune responses; the inflammation induced by pathological antibodies in the joints is independent of T cells and relies on innate immune components such as mast cells, neutrophils, and macrophages (46).

Dysbiosis in the gut microbiota triggers changes in arginine metabolism, leading to reduced serum polyamine levels, which hinder the differentiation and maturation of gut immune cells and subsequently regulate the immune response in AIH patients (23). Furthermore, increased levels of branched-chain amino acids (BCAAs) such as leucine, valine, and isoleucine in AIH patients help enhance both innate and adaptive immune responses and regulate gut barrier function through multiple critical signaling pathways (26). The mammalian Target of Rapamycin (mTOR) signaling pathway serves as a crucial nexus for nutrient sensing and metabolic signaling. The modulation of cellular growth and metabolism by BCAAs, predominantly leucine, occurs through the activation of mTOR complex 1 (mTORC1) (49). Under conditions of amino acid limitation, the brain-specific eIF2α kinase general control nonderepressible-2 (GCN2) is activated, leading to inhibited food intake, indicating that BCAAs regulate food consumption and metabolism via the GCN2 pathway. BCAAs also respond to cellular stress by regulating the phosphorylation of eIF2α, which in turn influences the autophagy process (50). BCAAs contribute to regulating gut barrier function by impacting immune cell activation and function. For instance, BCAAs interact with immunomodulatory molecules derived from the gut microbiota, regulating intestinal immune maturation and regulation. BCAAs modulate immune responses by influencing the regulation of natural killer T (NKT) cells (51). From an inflammatory response standpoint, BCAAs modulate inflammation by influencing macrophage polarization; leucine facilitates the transition from M1 (pro-inflammatory) to M2 (anti-inflammatory) macrophages through the mTORC1/LXR signaling pathway, thereby reducing inflammation (52). BCAAs regulate T cell activation and immune responses through specific amino acid transporters such as alanine-serine-cysteine transporter 2 (ASCT2) (53).

Secondary bile acids are ligands for the G-protein coupled bile acid receptor 1 (GPBAR1), which is typically expressed in NKT cells (54). In AIH, the reduced abundance of *Clostridium* leads to the lack of secondary bile acids (55), resulting in GPBAR1 inactivation, the inhibition of polarization from NKT10 cells to NKT cells, and a decrease in the secretion of the anti-inflammatory cytokine IL-10 (56). GPBAR1, also known as TGR5, is expressed in a variety of cell types, including metabolically active tissues, cholangiocytes, intestinal epithelial cells, brown adipose tissue, muscle, enteric and endocrine cells, primary sensory neurons, biliary cells, and the hypothalamus (57). These expression sites highlight the indispensable role of TGR5 in metabolic regulation, bile acid signaling, and energy expenditure (58). Notably, TGR5 expression in intestinal epithelial cells allows bile acids, as metabolites in the gut, to signal nutrient availability through TGR5 activation (54). TGR5 is highly expressed in cholangiocytes, where it enhances liver protection against bile acid overload by regulating the permeability of the biliary epithelium (59).

### Gut microbiota metabolites modulate microbiota composition through innate immunity activation in AIH

The intestinal microbiota and its metabolites play an indispensable role in the development of AIH through immune-microbial feedback loops (60, 61). Excessive gut-derived metabolites abnormally activate the innate immune system, subsequently leading to alterations in microbiota abundance associated with AIH (36). Firstly, the gut microbiota activate the immune system by influencing the differentiation and function of T cells. RORγt^+^ Th17 cells are crucial for mucosal defense; they accumulate in the gut in response to the microbiota and produce IL-17 cytokines. Specific symbiotic bacteria, such as segmented filamentous bacteria (SFB), induce the production of Th17 cells and exacerbate autoimmune responses in mice (62). This suggests that the gut microbiota directly activates specific T cell subsets, thereby regulating immune responses. Conversely, the role of the immune system in regulating the composition of the gut microbiota is also indispensable. Soluble lymphotoxin alpha (sLTα3), produced by RORγt^+^ innate lymphoid cells (ILCs), controls the T cell-dependent induction of IgA in the lamina propria by regulating T cells, thereby influencing the composition of the gut microbiota (63). Notably, bile acids are critical metabolites of the intestinal microbiota and influence the composition of the intestinal microbiota directly or indirectly by activating the innate immune system (64). Gut bacteria that regulate bile acid metabolism include *Bacteroides*, *Clostridium*, *Lactobacillus*, *Bifidobacterium*, and *Eubacterium*, which are commonly enriched in AIH patients (26).

## Gut microbiota influence T cell differentiation and function in AIH

**Figure 2:**
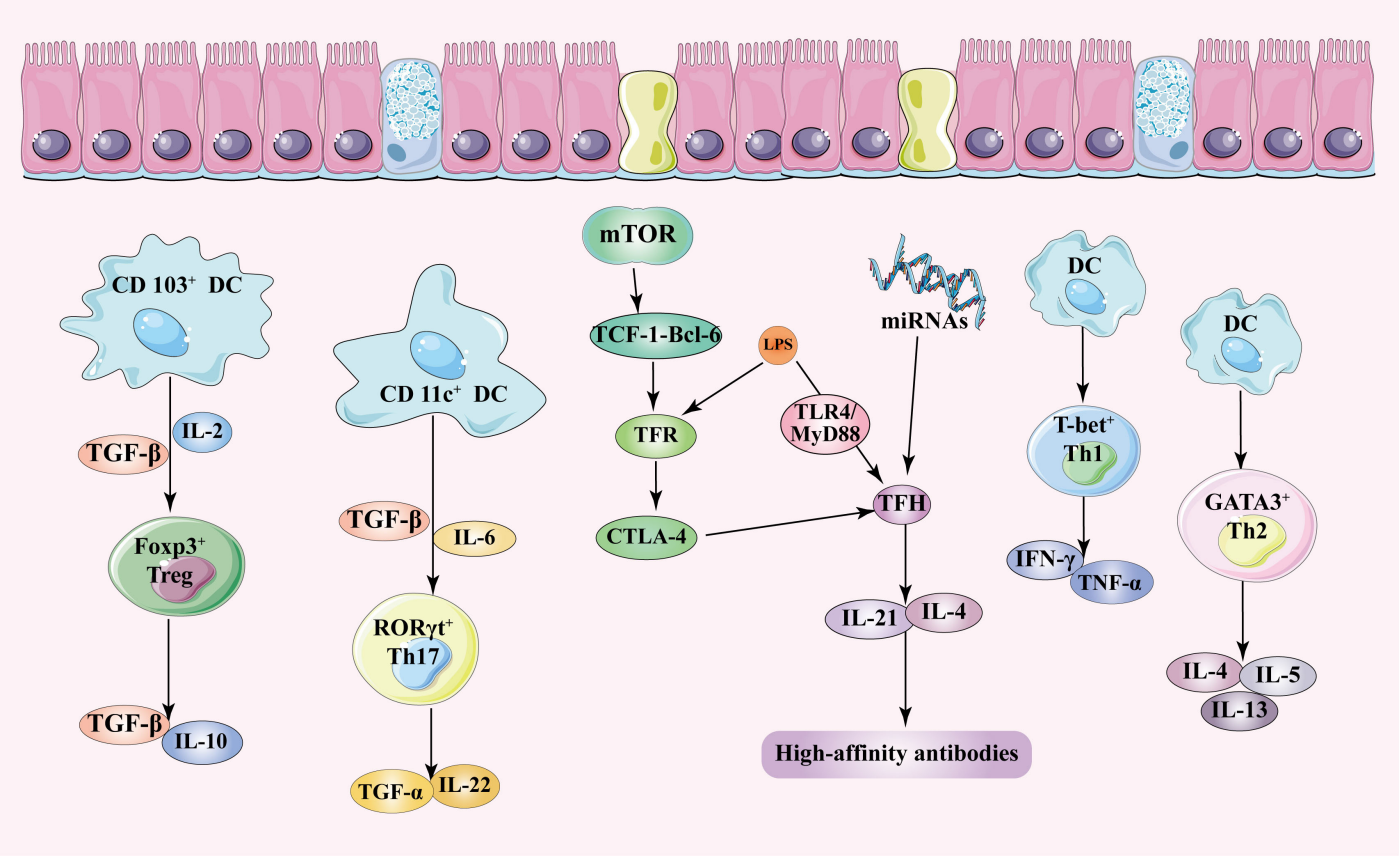
Signals from the gut microbiota lead to the differentiation of T cells into various subtypes. Many of these signals are mediated through epithelial cells or DCs. The gut microbiota promotes Treg generation through the presentation of antigens by goblet cells and CD103^+^ DCs, along with the secretion of high levels of TGF-β and IL-2. CD11c^+^ DCs detect gut microbiota and secrete TGF-β and IL-6, which drive Th17 polarization, leading to the secretion of TNF-α and IL-22. The gut microbiota also initiates TFR cell differentiation through mTOR-mediated activation of the TCF-1-Bcl-6 axis. Furthermore, the microbiota regulates the expression of microRNAs, which in turn regulates TFR cell differentiation and function. In AIH, elevated levels of LPS inhibit TFR cells and activate TFH cells via the TLR4/MyD88 signaling pathway, promoting the production of high-affinity antibodies by secreting IL-4 and IL-21. Gut microbiota is phagocytosed by DCs or stimulates them to release pro-inflammatory cytokines like IL-6 and TNF, leading to Th1 polarization. DCs produce IL-4 and TGF-β to facilitate Th2 cell differentiation. Th2 cells release cytokines such as IL-4, IL-5, and IL-13, which are associated with AIH. DC, dendritic cell; Treg, regulatory T cell; TGF-β, transforming growth factor-β; IL, interleukin; Th17, T helper 17; Th1, T helper 1; Th2, T helper 2; TFH, follicular helper T; TFR, follicular regulatory T; mTOR, mammalian target of rapamycin; TCF-1, T-cell-specific transcription factor 1; Bcl-6, B-cell lymphoma 6; LPS, lipopolysaccharide; CTLA-4, cytotoxic T-lymphocyte-associated protein 4; TLR4, toll-like receptor 4; MyD88, myeloid differentiation primary response gene 88.

Disruption of the gut microbiota activates autoreactive T cells, particularly CD4^+^ T cells and CD8^+^ T cells, thus exacerbating autoimmune reactions in target organs (65, 66). In AIH, APCs misrecognize self-antigens from the liver as foreign antigens, promote T cells, and induce their overactivation and tissue lesions (67). Hepatocytes, acting as unconventional APCs, enhance the autoimmune response in AIH through major histocompatibility complex (MHC) class II molecules on their surfaces (68). *In vitro* studies have shown that low concentrations of butyrate inhibit the proliferation of CD4^+^ T cells and CD8^+^ T cells (69). There exists a complex and dynamic interplay between the gut microbiota and CD4^+^ T cells, regulating adaptive and innate immunity during both homeostasis and inflammation (70). This interaction is crucial for maintaining health and disease states in the local gut and extraintestinal tissues. Activated CD4^+^ T cells are generally found in tissues with persistent microbial colonization, with the gastrointestinal tract being the most well-studied area. Interaction between the microbiota and self-recognition is common for CD4^+^ T cell populations and, if properly regulated, generally does not induce disease. Clearly, the microbiota plays an active role in this regulation, promoting the activation, polarization, and function of CD4^+^ T cells (71). Signals from microbes guide CD4^+^ T cells to polarize into four functionally distinct cell subtypes: T-bet^+^ Th1, GATA3^+^ Th2, RORγt^+^ Th17, and FOXP3^+^ Tregs. Numerous signals are conveyed through epithelial or dendritic cells (DCs) (1). Aberrant composition and metabolic imbalance of the gut microbiota contribute to the pathogenesis of AIH by disturbing immune homeostasis, such as the imbalance between Tregs/Th17, T follicular regulatory (TFR)/T follicular helper (TFH), and Th1/Th2 immune cells (**Figure 2**). Additionally, they also regulate the effector of NKT cells and γδT cells.

### Treg/Th17 cells

Tregs and Th17 cells are pivotal subsets of T cells within the immune response framework. Tregs function mainly as immunosuppressants, aiding in the preservation of immune tolerance and preventing autoimmune maladies. Conversely, Th17 cells are strongly associated with inflammation and the progression of autoimmune diseases (72). A hallmark of autoimmune disorders is the dysregulated equilibrium between Tregs and Th17 cells (73). The gut microbiota modulates the differentiation and function of Tregs and Th17 cells by producing metabolic byproducts such as SCFAs. Notably, butyrate, a type of SCFAs, has been shown to promote the differentiation and functionality of Tregs while simultaneously restraining the development of Th17 cells (72, 74). The gut microbiota interacts with immune cells through pattern recognition receptors (PRRs), impacting the differentiation of Tregs and Th17 cells. For instance, certain components of gut microbiota activate TLRs on DCs, subsequently influencing the differentiation and functionality of Tregs and Th17 cells. The gut microbiota influences the balance of Tregs and Th17 cells by modifying the functionality of DCs. In cases of *Helicobacter pylori* infection, DC-induced Treg skews aid in the immune evasion of *Helicobacter pylori* while concurrently diminishing the immune response of Th17 cells (75). By influencing T cell metabolic pathways, the gut microbiota plays a role in the differentiation process of Tregs and Th17 cells. For example, the expression and inhibition of PDHK1 in Th17 cells regulate the Th17/Tregs ratio, thereby regulating immune responses and the progression of autoimmune disorders (76). The gut microbiota also sway the production of cytokines such as IL-6, which are instrumental in the differentiation of Tregs and Th17 cells. IL-6 is a key factor for Th17 cell differentiation, whereas Tregs differentiation relies on transforming growth factor-β (TGF-β) and IL-2 (77). Additionally, the gut microbiota regulates Tregs and Th17 cell differentiation by influencing TGF-β activation, a shared factor crucial for maintaining the balance between these cell types (78). In summary, the gut microbiota modulates the balance between Tregs and Th17 cells through various mechanisms, which include the production of metabolic byproducts, direct interaction with immune cells, regulation of cytokine production, and influence on key transcription factors involved in T cell differentiation. These mechanisms collectively regulate the development and progression of autoimmune diseases.

In patients with AIH, dysbiosis of the gut microbiota alters the metabolism of gut contents, increasing the proportion of Th17 cells while reducing the proportion of Tregs (46, 79). In rodent models of inflammation, administration of SCFAs suppresses inflammatory diseases by increasing the number of Tregs (80, 81). Butyrate promotes Tregs differentiation by inhibiting histone deacetylase and promoting acetylation of histone H3 of Foxp3 (82). Studies have shown that the aberrant Th17 cell response in AIH is associated with altered aryl hydrocarbon receptor (AHR) signaling, leading to reduced responsiveness of Th17 cells to AHR activation. Furthermore, AHR activation induces the upregulation of ectonucleoside triphosphate diphosphohydrolase 1 (CD39). CD39 is an extracellular enzyme that hydrolyzes ATP to produce the immunosuppressive adenosine, and the imbalance between Tregs and Th17 is closely related to low levels of CD39 (83). The increase in IL-17 drives newly generated Tregs to polarize into pro-inflammatory Tregs with immune activation functions, impairing the function of Tregs. This promotes immune cell infiltration and liver damage, further leading to liver inflammation and fibrosis (67). Th17 cells exacerbate liver inflammatory damage and immune attacks by promoting the secretion of pro-inflammatory cytokines such as TNF-α and IL-22 (84). Conversely, Tregs maintain immune homeostasis by inhibiting the activation of immune effector cells through the release of TGF-β and IL-10, or by interacting with DCs to inhibit their function (85, 86). Therefore, the increase in Th17 cells and decrease in Tregs induced by gut microbiota dysbiosis in AIH patients disrupts immune homeostasis, exacerbates inflammatory damage, and promotes disease progression.

### Th1/Th2 cells

Th1 and Th2 cells are two primary subsets of CD4^+^ T cells, essential in modulating adaptive immune responses and potentially inducing certain types of autoimmune diseases. Th1 cells predominantly secrete interferon-γ (IFN-γ) and TNF-α and are involved in cell-mediated immunity and defense against intracellular pathogens. In contrast, Th2 cells release cytokines such as IL-4, IL-5, and IL-13, which are associated with allergic reactions and anti-parasitic immunity (87). During the differentiation of Th1/Th2 cells, SCFAs produced by the gut microbiota, such as butyrate, promote the generation of Treg cells, thereby inhibiting the differentiation of Th1 and Th2 cells (88). The gut microbiota directly interacts with immune cells in the gut-associated lymphoid tissue (GALT), influencing the Th1/Th2 balance. For instance, certain gut microbes activate DCs to secrete cytokines favoring a Th2 response, like IL-4, thereby promoting Th2 cell differentiation (88). The gut microbiota is involved in various metabolic pathways, including those of carbohydrates and fats, which regulate Th2 cell differentiation. Several studies suggest that metabolic pathways associated with Th2 cells are more abundant in individuals with obesity and asthma (89). The gut microbiota enhances the development and function of Treg cells, which secrete cytokines like IL-10 and TGF-β to suppress excessive Th1 and Th2 responses, hence maintaining immune tolerance (88). The gut microbiota influence host signaling pathways, such as the JAK-STAT pathway, which plays a crucial role in Th1/Th2 differentiation (90). In summary, the gut microbiota regulates the Th1/Th2 balance through multiple mechanisms, impacting the onset and progression of autoimmune diseases. These mechanisms include regulating T cell differentiation, interacting with immune cells, participating in metabolic pathways, enhancing Treg cell function, and influencing signaling pathways.

### TFR/TFH cells

TFH cells are crucial for B cell activation, antibody production, class switch recombination, and affinity maturation (91). TFH cells drive differentiation by expressing Bcl-6 and assisting B cell regions (92). TFR cells, a subset of regulatory T cells, control antibody production by inhibiting TFH-mediated help. TFR cells fine-tune antibody responses and prevent excessive immune reactions through their suppressive functions (93). The gut microbiota regulate TFR cell differentiation by regulating signaling pathways. For instance, mTORC1 (mammalian target of rapamycin complex 1) initiates TFR cell differentiation during immune responses or infections by activating the TCF-1-Bcl-6 axis (94). The gut microbiota indirectly influences TFR cells by regulating TFH cell functions. TFH cells are key mediators of germinal center (GC) formation, whereas TFR cells suppress TFH-mediated GC responses (91). The gut microbiota regulates TFH and TFR cells by influencing the expression of immune regulatory molecules. The microbiota has the potential to modulate the expression of pro-inflammatory microRNAs (miRNAs), like miR-155, and anti-inflammatory miRNAs, like miR-146a, by activating host cell signaling pathways through metabolites or pathogen-associated molecular patterns (PAMPs). This regulation is crucial for the generation of TFH cells and influences the differentiation and function of TFR cells (91). For example, dysbiosis in the gut microbiota leads to the upregulation of pro-inflammatory miRNAs, like miR-21, which suppress TFR cell function and enhance TFH cell activity. This imbalance potentially exacerbates the production of autoantibodies during GC reactions (95). In summary, the gut microbiota regulates the balance between TFR and TFH cells through various mechanisms, including influencing TFR cell differentiation, TFH cell function, and the expression of immune regulatory molecules.

In the pathogenesis of AIH, the abnormal selection of high-affinity autoreactive plasma cells in the GC plays a central role. TFH cells provide signals for B cells survival and differentiation through the expression of inducible T-cell co-stimulator (ICOS) and CD40 ligand (CD40L). Additionally, TFH cells promote the production of high-affinity antibodies by secreting soluble factors such as IL-4 and IL-21. Thus, uncontrolled proliferation of TFH cells leads to excessive production of autoreactive B cells and autoantibodies, triggering an autoimmune response (96). Studies have shown that TFR cells express proteins typical of both TFH cells (ICOS and PD-1) and Tregs (CD25 and CTLA-4). Consistent with this, the study indicates that TFR cells are thymic-derived Tregs that migrate to the follicles in a TFH cell-dependent manner. Similar to TFH cells, levels of PD-1^+^ TFR and ICOS^+^ TFR cells are significantly elevated in AIH patients, suggesting that activated TFR cells suppress aberrant B cell activation and differentiation by providing negative signals (97). Furthermore, AIH patients show reduced expression of CTLA-4 in TFR cells; as CTLA-4 is crucial for maintaining immune tolerance and homeostasis, its reduction enhances cell-mediated immune responses and antibody production (98, 99). In summary, the increase of PD-1 and ICOS and the decrease of CTLA-4 on TFR cells involve regulating B cell responses in the pathogenesis of AIH. Elevated LPS in AIH disease models inhibits TFR cells and activates TFH cells through the TLR4/MyD88 signaling pathway (100, 101). The overactivation of TFH cells is closely associated with hypergammaglobulinemia, which accelerates the immunopathological process of AIH (102). TFR cells indirectly inhibit the activation of TFH cells by recognizing CTLA-4, thereby reducing autoantibody production. Therefore, the imbalance between TFR and TFH cells leads to disrupted immune homeostasis and excessive autoantibody secretion, participating in the immunopathological process of AIH (103).

### NKT cells

Studies have shown that the microbiota regulates susceptibility to liver injury through the Fas/FasL pathway, and liver injury mediated by NKT cells largely involves this pathway. This suggests that the impact of the microbiota on liver injury is closely related to the function of NKT cells (104). In the mechanism by which pathogenic bacteria exacerbate Con-A-induced liver injury, NKT cells play a key role, and their activation partially depends on IL-12 produced primarily by DCs (105). In the Con-A-induced fulminant hepatitis model, similar to the situation in AIH patients, NKT cells in the liver can be activated by gut pathogens through two pathways. One possible pathway is that intestinal pathogens trigger the activation of gut DCs, which then migrate to the liver via Peyer’s patches (PPs), promoting the activation of NKT cells. Another possible pathway involves a large number of translocated intestinal antigens that first enter the liver and activate hepatic DCs, thereby activating NKT cells (105). The activated NKT cells further stimulate Kupffer cells and recruit macrophages, which secrete large amounts of inflammatory cytokines, subsequently initiating repair responses through activated hepatic stellate cells (HSCs), including hepatocyte regeneration and fibrosis (106). These responses collectively exacerbate the progression of liver inflammatory damage and fibrosis in AIH patients.

### γδT cells

γδT cells are abundant in the human liver but represent a small proportion of immune cells in peripheral blood (107). As unconventional T lymphocytes, γδ T cells function by expressing the γδ T cell receptor (TCR) and are independent of antigen presentation by MHC. They are capable of recognizing MHC class I chain-related antigens A and B (MICA and MICB) as well as non-peptide metabolites produced by the isoprenoid biosynthesis pathway. Even in the absence of TCR stimulation, γδ T cells rapidly act upon activation by cytokines, allowing them to respond earlier than αβ T cells. In the mucosal barrier, microbial communities provide chronic stimulation to γδ T cells, thereby constraining their effector functions. This regulatory role is crucial for maintaining immune homeostasis at the mucosal barrier and for preventing excessive immune responses (108). The gut microbiota also influences the development and maturation of γδ T cells. Studies have shown that the presence of the gut microbial community is essential for the maturation and functional expression of γδ T cells, particularly during the early stages of life (109). Additionally, the gut microbiota regulates the tissue residency characteristics of γδ T cells. Similar to tissue-resident memory T cells, the gut microbiota modulate the transcriptional programs associated with the tissue residency of γδ T cells (110). The regulatory influence of the gut microbiota on γδ T cells also manifests in the heterogeneity of γδ T cells. Different subsets of γδ T cells respond differently to the gut microbiota, resulting in functional and effector diversity among γδ T cells (110). In summary, the gut microbiota regulates γδ T cells through multiple mechanisms, including directly regulating their effector functions, influencing their development and maturation, modulating their tissue-residency characteristics, and contributing to their heterogeneity.

The microbiota is crucial for maintaining the homeostasis of hepatic γδT17 cells, potentially involving lipid antigens provided by the microbiota. Liver cells present these antigens through CD1d, activating γδ T cells and inducing the production of IL-17A. Study shows that activated γδT17 cells exhibit pro-inflammatory and anti-infective abilities (111). A recent study shows that certain bacterial groups significantly stimulate the proliferation of colonic lamina propria γδT17 cells. For example, *Bifidobacterium* and *Bacillus* enhance barrier function by promoting the expression of TLR2 on γδ T cells. However, *in vitro* experiments show that only *Bacillus* promotes the expression of TLR2 and IL-17, while *Bifidobacterium* does not have this effect. Additionally, the study found a positive correlation between *Bifidobacteriaceae* and γδT17 cells, while *Prevotellaceae*, *Rhodospirillaceae*, and *Flavobacteriaceae* showed a negative correlation with γδT17 cells in the gut (112).

## T cells induce gut microbiota abundance and followed AIH progression

Although the gut microbiota has an indispensable impact on the function of the mucosal immune system, particularly the functions of T lymphocytes and B lymphocytes (113), whether lymphocytes can, in turn, regulate the microbiota remains insufficiently studied. Recently, scholars have begun to focus on the relationship between T cell subsets and variations in the relative abundance of intestinal bacteria. Kierasinska M et al. explored whether dysregulation of the gut microbiota is a precursor or a consequence of the development of autoimmune diseases. However, it is well established that dysbiosis is associated with an increase in pro-inflammatory lymphocytes, particularly within the Th17 cell population. This suggests that T cells play a role in modulating changes in the gut microbiota, especially in the context of autoimmune disorders such as AIH (114).

### The impact of T cells on gut microbiota composition

**Figure 3:**
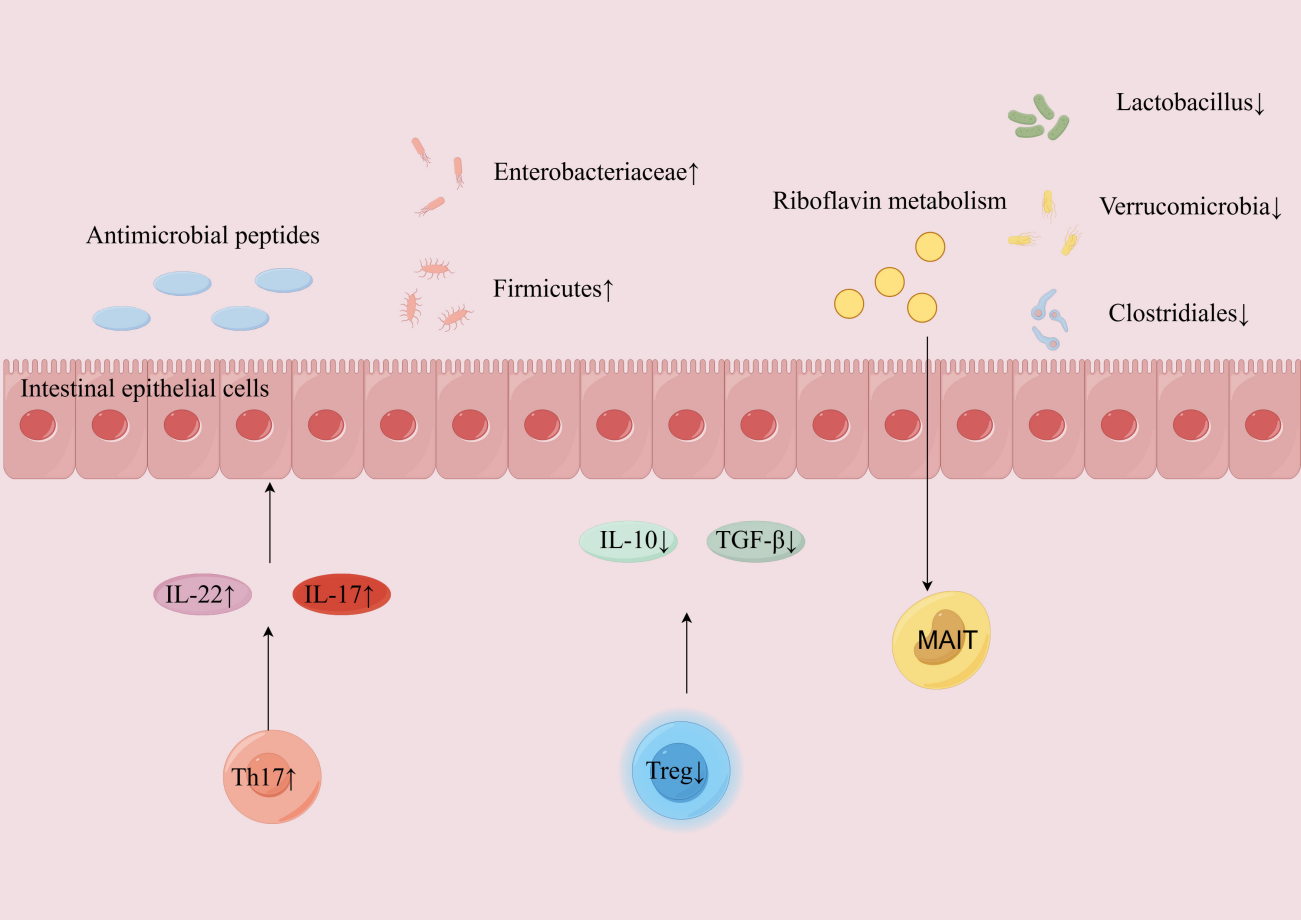
T cells modulate the composition and abundance of gut microbiota through the secretion of cytokines in AIH. The hyperactivation of Th17 cells leads to an increased secretion of pro-inflammatory cytokines such as IL-17 and IL-22, which stimulates epithelial cells to release antimicrobial peptides and triggers local inflammation. Concurrently, a dysfunction in Treg cells results in decreased secretion of anti-inflammatory cytokines like IL-10 and TGF-β, disrupting intestinal immune tolerance. This imbalance exacerbates gut dysbiosis, promoting the proliferation of pathogenic bacteria such as *Enterobacteriaceae*, while inhibiting the growth of beneficial bacteria like *Lactobacillus*. MAIT cells recognize riboflavin metabolites and monitor disturbances within the intestinal microbiome. AIH, autoimmune hepatitis; Th17, T helper 17; IL, interleukin; Treg, regulatory T cell; TGF-β, transforming growth factor-β; MAIT, mucosal-associated invariant T.

T cells can respond to changes in the gut microbiota and regulate immune responses by secreting cytokines like interferon-gamma and IL-17, which regulate the composition of the gut microbiota (**Figure 3**) (115). Specific gut microbes induce specific T-cell responses, potentially regulating the abundance and composition of the gut microbiota. For example, T cell response patterns are attributed to changes in the abundance of certain strains within the gut microbial community (116).

Tregs secrete anti-inflammatory cytokines, such as IL-10 and TGF-β, playing a crucial role in modulating immune responses and promoting immune tolerance, consequently reducing immune attacks against intestinal microbial antigens (117). There is a positive correlation between the expression of Foxp3 in Tregs and specific gut microbiota like the order *Clostridiales* and the phylum *Verrucomicrobia* (118). It was revealed that the gut microbiota of DEREG mice showed multidimensional separation after Tregs depletion compared to pre-depletion and wild-type mice samples. Remarkably, following Tregs depletion, there was increased relative abundance of the phylum *Firmicutes* associated with intestinal inflammation (119).

Th17 cells produce cytokines such as IL-17 and IL-22, which play a crucial role in stimulating epithelial cells to secrete antimicrobial peptides. These peptides are vital for the control of harmful bacteria proliferation and balance of the gut microbiota (120). Beyond their direct impact on the gut flora, Th17 cells also influence the intestinal microbial environment through interactions with other immune cells. For instance, modulating the Th17/Tregs balance augmented the abundance of beneficial bacteria while reducing the prevalence of harmful counterparts (121).

It was revealed that modulating Th1 cell-related signaling pathways, such as the TLR4-myD88/TRIF pathway, altered the distribution of gut microbiota, thus exerting a protective effect against autoimmune diseases like T1D (122). Adjusting Th1 cells ameliorated the imbalance of gut microbiota, alleviating the symptoms of IBD (123). In colitis models, the modulation of Th1/Th2 and Tregs/Th17 cell balance, alongside the inhibition of the NLRP3 inflammasome, remodeled the gut microbiota, providing new therapeutic strategies for patients with colitis (124). Th1 and Th2 cells regulated immune responses by influencing the composition of gut microbiota, thereby participating in the progression of AIH (125, 126).

Additionally, inflammation induces the composition and metabolism of the microbiome, but how the host monitors and responds to alterations remains unclear. Studies have described a protective mechanism where mucosal-associated invariant T (MAIT) cells detect microbiome-derived metabolites resulting from inflammation and promote tissue repair (127). MAIT cells recognize microbiome-derived metabolites, particularly those associated with riboflavin (vitamin B) metabolism. By detecting these metabolites, MAIT cells play a crucial role in monitoring imbalances within the gut microbiome (128).

### Alterations in the thymic T cell compartment influence gut microbiota distribution

Abnormalities in the thymic T cell compartment not only promote the development of AIH but also regulate the distribution of the gut microbiota. The thymic T cell compartment regulates autoimmune susceptibility by inducing complex changes in the gut microbiota and is a potent genetic determinant. Improper selection of T cells in the thymus leads to changes in the gut microbiota, exacerbating organ-specific autoimmunity and the worsening of AIH. The study by Monica Centa et al. provides evidence that endogenous T cell compartment characteristics regulate the formation of the gut microbiota, resulting in organ-specific autoimmunity. The study reveals a connection between abnormal T cells and altered gut microbiota distribution in Traf6ΔTEC mice, due to the lack of mTECs, emphasizing the interaction between T cells and the gut microbiota. In Traf6ΔTEC mice, changes in antigen presentation lead to specific changes in gene mutations within the T cell repertoire, which in turn shapes a unique gut microbiota (129). Studies have demonstrated that the absence of TOX profoundly impacts the thymic microenvironment, particularly during the maturation of mTECs. Furthermore, the loss of TOX significantly promotes the differentiation and formation of IL-17A-secreting γδ T cells, known as γδT17 cells. The abnormal increase of these cells is considered a critical factor contributing to the development of fatal AIH (130). However, whether the increasing γδT17 cells directly damage liver tissues or indirectly promote AIH by altering the composition and distribution of the gut microbiota remains to be investigated.

## Promising therapy of AIH based on the mechanisms of immune-microbial microenvironment

Currently, there is no effective treatment strategy for AIH. Glucocorticoids alone or in combination with azathioprine are the main treatments for AIH. These therapies effectively alleviate symptoms in part of AIH patients and extend their survival time (131). However, other patients remain unable to achieve symptom relief with standard therapies or develop medicine resistance. In addition, these drugs probably cause side effects such as osteoporosis, bone marrow suppression, central obesity, and liver function damage (132). Given the significant role of the gut microbiota in the occurrence and development of AIH, therapies targeting the gut microbiota are considered a new direction for AIH treatment. These methods include probiotics (133), fecal microbiota transplantation (FMT) (134), and certain drugs targeting gut microbiota-related signaling pathways (135, 136). Studies have suggested that these three strategies have demonstrated the potential to reduce autoimmune hepatitis symptoms in AIH models, suggesting that microbiota-targeted therapies offer new hope for AIH patients.

In recent years, Chimeric Antigen Receptor (CAR) -T cell therapy has increasingly drawn attention. Its basic principle involves the ex vivo genetic modification of a patient’s T cells to express specific chimeric antigen receptors. These modified T cells are then reintroduced into the patient’s body, enabling them to recognize and destroy tumor cells expressing the corresponding antigen. Although CAR-T cell therapy has made significant progress in cancer treatment, its application in autoimmune diseases is still in the early study stage. CAR-T cell therapy significantly reduces the number and functionality of B cells by profoundly depleting them within tissues, thereby decreasing the production of autoantibodies. Additionally, it inhibits the antigen-presenting function of B cells, which aids in disrupting pathogenic immune cyclic responses. Furthermore, it reduces cytokine production by B cells, diminishing inflammatory responses. In autoimmune diseases such as multiple sclerosis, B cells trigger inflammatory T cell responses by presenting self-peptides. By depleting B cells, CAR-T cell therapy indirectly reduces T cell activation (137). Potential applications of CAR-T cell therapy in autoimmune diseases include: 1) Modulating autoreactive T cells: through genetic modification, T cells express receptors that inhibit autoreactive T cells to alleviate autoimmune responses. 2) Inducing immune tolerance: modified T cells express receptors that promote immune tolerance, preventing the immune system from attacking self-tissues. 3) Targeting specific immune cells: by specifically recognizing and modulating receptors on certain immune cells such as Th17 and Treg cells, the progression of autoimmune diseases be influenced (138). Recent studies have shown that CAR-T cell therapy targeting CD19 is safe and effective for treating certain autoimmune diseases, such as systemic lupus erythematosus, systemic sclerosis, and idiopathic inflammatory myopathies (139). However, numerous challenges remain in the treatment of autoimmune diseases with CAR-T cell therapy, including precise targeting of pathological tissues, avoiding damage to normal tissues, and controlling the activity of modified T cells (137). Additionally, further studies will be needed to verify their long-term safety and efficacy. It is noteworthy that studies and applications of CAR-T cell therapy are in progress, and it is expected to provide innovative strategies and methods for the treatment of autoimmune diseases in the future. Considering the application of T cells modified by intestinal microbiota and their metabolites is inspired by natural reactions, there will possibly be more advantages than CAR-T therapies alone in terms of safety, after considerable effectiveness evaluation.

## Conclusion

The gut microbiota and the host maintain a mutually beneficial symbiotic dynamic balance, playing a crucial role in numerous physiological and pathological processes, such as maintaining mucosal immune homeostasis and regulating the development of autoimmune and inflammatory diseases. The gut-liver axis profoundly influences the normal physiological functions of the liver through the dynamic changes in the gut microbiota and their metabolic products. In recent years, increasing studies have focused on the potential interaction between the gut microbiota and AIH. When the gut microbiota shifts from an anaerobic to an aerobic environment, it provokes immune responses and alters metabolites, including imbalances between Tregs/Th17, TFR/TFH, and Th1/Th2 cells, activation of NKT cells, and reductions in SCFAs and secondary bile acids, ultimately leading to the disruption of the gut barrier and immune homeostasis. Notably, the critical role of γδT cells in the pathogenesis of AIH is gradually gaining recognition. However, studies into the interaction between the gut microbiota and γδT cells remain insufficient.

During the progression of AIH, alterations in the gut microbiota modulate the differentiation and function of T cells, leading to an imbalance among T cells. This imbalance further exacerbates the inflammatory response. Conversely, the imbalance in T cells regulates the abundance of the gut microbiota, creating a vicious cycle that further exacerbates AIH. Although it remains unclear whether dysbiosis of the gut microbiota or T-cell abnormalities represent the initial trigger in the development of AIH, it is undeniable that the interaction between the gut microbiota and T cells plays an indispensable role.

Currently, targeting the gut microbiota as a treatment for AIH has become a study highlight. The importance of CAR-T cell therapy in the treatment of autoimmune diseases has been widely recognized. Although this therapy’s effectiveness in AIH treatment has not been fully verified, it could potentially open new avenues for AIH treatment. Clarification of the mutual regulatory effects between the gut microbiota and T cells is crucial for revealing the pathogenesis underlying AIH and identifying another effective therapeutic target.
